# Boronate Complex Formation with Dopa Containing Mussel Adhesive Protein Retards pH-Induced Oxidation and Enables Adhesion to Mica

**DOI:** 10.1371/journal.pone.0108869

**Published:** 2014-10-10

**Authors:** Yajing Kan, Eric W. Danner, Jacob N. Israelachvili, Yunfei Chen, J. Herbert Waite

**Affiliations:** 1 Jiangsu Key Laboratory for Design and Manufacture of Micro-Nano Biomedical Instruments, and School of Mechanical Engineering, Southeast University, Nanjing, China; 2 Biomolecular Science and Engineering, University of California Santa Barbara, Santa Barbara, California, United States of America; 3 Department of Chemical Engineering, University of California Santa Barbara, Santa Barbara, California, United States of America; Consejo Superior de Investigaciones Cientificas, Spain

## Abstract

The biochemistry of mussel adhesion has inspired the design of surface primers, adhesives, coatings and gels for technological applications. These mussel-inspired systems often focus on incorporating the amino acid 3,4-dihydroxyphenyl-L-alanine (Dopa) or a catecholic analog into a polymer. Unfortunately, effective use of Dopa is compromised by its susceptibility to auto-oxidation at neutral pH. Oxidation can lead to loss of adhesive function and undesired covalent cross-linking. Mussel foot protein 5 (Mfp-5), which contains ∼30 mole % Dopa, is a superb adhesive under reducing conditions but becomes nonadhesive after pH-induced oxidation. Here we report that the bidentate complexation of borate by Dopa to form a catecholato-boronate can be exploited to retard oxidation. Although exposure of Mfp-5 to neutral pH typically oxidizes Dopa, resulting in a>95% decrease in adhesion, inclusion of borate retards oxidation at the same pH. Remarkably, this Dopa-boronate complex dissociates upon contact with mica to allow for a reversible Dopa-mediated adhesion. The borate protection strategy allows for Dopa redox stability and maintained adhesive function in an otherwise oxidizing environment.

## Introduction

**Figure 1:**
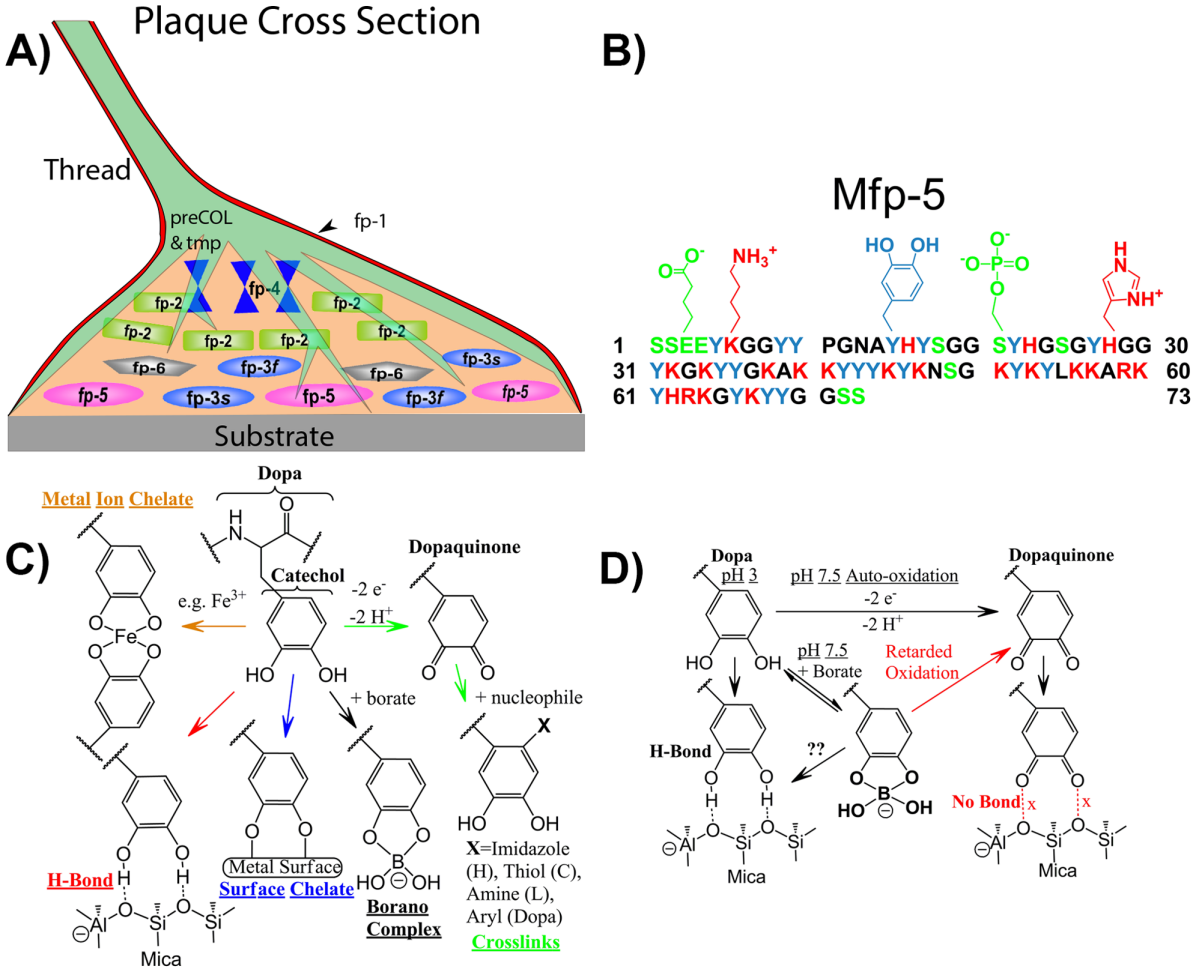
A schematic overview of Dopa-proteins and Dopa in mussel adhesion. (A) Schematic cross-section of an adhesive plaque with mussel foot protein distribution. (B) Sequence of Mfp-5 highlighting distribution of Dopa (blue) and charged residues. (C) The versatile reactivity of Dopa in adhesion, cohesion and borate complexation. (D) The complexation of Dopa and borate to form an oxidation resistant Dopa-boronate species at pH 7.5 has critical implications for adhesion. Dopaquinone, which normally forms from the 2-electron oxidation of Dopa at pH 7.5 in the absence of borate, is unable to bond mica.

The holdfasts of marine mussels are recognized for their durable adhesion to a variety of solid surfaces under high-flow, wet, and saline conditions – a much prized though elusive capability for a wide range of potential applications. Holdfasts consist of byssal threads whose distal extremities end in adhesive plaques. The adhesive plaques contain mussel foot proteins (Mfps) that include the amino acid 3,4-dihydroxyphenylalanine (Dopa) (Fig 1a–b) [1]. Dopa is critical for Mfp adhesion and Dopa, or the catecholic functional group of Dopa, is increasingly adopted for synthetic bio-inspired adhesives, coatings, scaffolds, sensors and gels [2]–[4].

Some of the appeal of Dopa and catechol functionalities stems from their versatile reaction chemistry (Fig. 1c). Under reducing conditions, Dopa binds to surfaces through bidentate metal coordination [5]–[7] or hydrogen bonding [4], [8]. Hydrogen bonding, metal ion coordination [9] and probably π-cation interactions [10] contribute significantly to cohesion as well. Under oxidizing conditions (chemical or at elevated pH), Dopa is converted to Dopaquinone. Permanent chemical bonds involving Schiff-base-, Michael-type additions or aryl-aryl coupling [4], [11]–[14] occur between Dopaquinone and neighboring Dopa, lysine, histidine or cysteine residues [11]–[13], [15]. This covalent bonding mediates curing of adhesive plaque proteins.

The chemical versatility of Dopa, however, is an asset only if its reactivity can be precisely controlled. This is not usually the case. For example, auto-oxidation of Dopa in Mfps at pH 7.5, results in the loss of adhesion to both mica and titanium [3], [4], [16]. Single molecule AFM studies of Dopa on TiO_2_ measured a separation force of 800 pN, which decreased to 180 pN upon oxidation to Dopaquinone [7]. Oxidation can also result from redox exchange with metal ions (e.g. Fe^III^ [17]), or from a pH dependent oxidation reaction with O_2_ [18]. Mussels limit Dopa oxidation in Mfps by the co-secretion of acid (pH ≤5) and anti-oxidant proteins [3]. Similarly in purified Dopa-containing proteins and catechol-functionalized polymers, oxidation is controlled by use of low pH buffers [18], [19], antioxidants [3] and electron withdrawing groups [16].

Mussel foot protein 5 (Mfp-5) contains ∼30 mol% Dopa, the highest level known in a protein. Mfp-5 functions as an interfacial adhesive in the mussel adhesive plaque (Fig. 1b) [4], [12], [20], and characterization by the surface forces apparatus (SFA) showed Mfp-5 to have an adhesion energy (∼14 mJ/m^2^) to mica that surpassed adhesion between well-oriented biotin/streptavidin surfaces [4]. Strong adhesion was observed when Dopa was kept reduced but exposure to neutral pH reduced adhesion by>90% [4].

The present study examines the reaction between Dopa and borate to form Dopa-boronate complexes, which resist oxidation while allowing for Dopa-mediated adhesion. At pH 7, borate complexes Dopa in a reversible, pH-dependent manner, and the resulting boronate complex formed is a negatively charged ion (Fig. 1c–d). The stability constant (K_s_  = 2.05×10^4^) of the catechol-boronate complex has been investigated in some detail [21]–[24]. Borate complexation to Dopa is used in the purification of mussel foot proteins [4], [20], [25] and in pH responsive synthetic systems [26]–[28]. We show that borate can bind and protect Dopa-rich Mfp-5 from oxidation, and then on contact with mica, the Dopa-boronate complex exchanges borate for direct Dopa adhesion to mica. Borate addition thus allows for strong, but reversible Dopa mediated adhesion at pH regimes that would otherwise lead to detrimental oxidation.

## Methods

### Mfp-5 Purification

Mussel foot protein-5 (Mfp-5) was extracted from *Mytilus edulis* (L. 1758) similarly to previous reports [4], [20]. In short, *M. edulis* feet were dissected and phenol glands pooled. Five grams of pooled glands were homogenized sequentially in A) 5% acetic acid (HAc), 1 µM leupeptin/pepstatin B) 5 v/v% HAc, 8 M urea C) 5% HAc, 6 M guanidinium hydrochloride. Homogenized solutions were centrifuged at 40,000 g for 45 min and the pellet was homogenized in the next solution. Sample were kept <4°C at all times. Supernants from B and C were stirred with 33% (w/v) ammonium sulfate for 30 min and centrifuged. Both supernants were subjected to dialysis in 4 L of 0.1% perchloric acid and 0.1 M borate (pH 8.2). Precipitated proteins were separated with HPLC (C-8) and Shodex (KW-802.5) chromatography which removed all salts and borate molecules used in purification. Purification was variable and Mfp-5 could be detected in the B and C steps, or both. Once extracted, Mfp-5 purity was checked by matrix assisted laser desorption ionization mass spectroscopy and by acid-urea polyacrylamide gel electrophoresis (5% acetic acid and 8 M Urea) [29]. Purified protein was suspended in 0.1 M acetic acid, concentration determined by UV-Vis, and diluted to 15 µg/mL for deposition onto mica. Mfp-5 solutions were aliquoted and stored at −80°C until use.

### Surface Force Apparatus

The surface forces apparatus (SFA 2000, SurForce LLC, Santa Barbara, CA) was used to measure the force-distance profiles of Mfp-5 layers compressed between two mica surfaces. Technical details of the SFA setup are available [30]–[32]. In this work, two freshly cleaved mica sheets were mounted in the SFA chamber in a cross-cylinder geometry. Distance of zero (D = 0) represents the contact between bare mica surfaces in air. Mfp-5 was adsorbed onto one mica surface from a 0.1 M acetic acid (HAc) droplet containing 300 ng of protein for 20 min. The treated and untreated mica surfaces were then rinsed with 0.1 M HAc. All measurements were done in solution i) pH 3, 0.1 M HAc, 0.25 M potassium nitrate (KNO_3_) or ii) pH 7.5, 0.1 M phosphate, 0.25 M KNO_3_, titrated with NaOH. For borate protection experiments, either 0.1 M of borate or phenylboronic acid (PBA) was added to the relevant buffer. Periodate oxidation involved injection of 20 µL of 1 mM sodium periodate into the working solution. The adhesion energy, E_ad_, was determined by 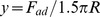 (JKR theory), where *F*_ad_ is the measured adhesion force and *R* is the surface radius [32]. Surfaces were brought into contact and held at a load of 5–10 mN/m. The term ‘hard wall’ refers to the thickness of the incompressible protein layer and approximates the protein hydration diameter. ‘Short contact’ refers to <1 min that mica is in contact with the protein film before a separation force is applied. Shown force runs are representative of multiple experiments. An overview of how to read SFA data is shown in Fig. S5.

### X-Ray Photoelectron Spectroscopy (XPS)

Pieces of freshly cleaved 1 cm^2^ mica were exposed to various solutions i) 0.1 M borate, 0.1 M PBS, pH 7.5 ii) 0.1 M borate, 0.1 M HAc, pH 3 iii) 0.1 M HAc, pH 3, 300 pg Mfp-5 iv) 0.1 M borate, 0.1 M PBS, pH 7.5, 300 pg Mfp-5. Solution droplets were in contact with mica for 30 minutes. Wafers were briefly rinsed with DI water to remove unbound salts and proteins. The samples were then dried and measured with a Kratos Axis Ultra X-ray Photoelectron Spectroscopy (XPS) system using charge neutralization. Each measurement used a pass energy of 160, 150 ms dwell time and averaged two sweeps. Data shown are representative of 3× replicates measured each at 2 or more locations.

### SIMS Measurements of Boron in Mussel (Fig. S1)

#### Tissue Preparation

For foot sections, local *Mytilus californianus* mussels were collected from the Goleta Pier, Santa Barbara, Ca (Under a California Department of Fish and Wildlife Scientific Collecting Permit (DFW SCP)). Mussels were euthanized, feet were amputated and fixed in 4% formaldehyde for 6 hours at RT. For plaque sections, plaques were deposited by *M. californianus* mussels placed on 10”–14” Plexiglas sheets. Fresh plaques were collected daily. Tissue and plaque sections were performed on a Leica CM1850 cryostat.

#### Light Microscope (LM)

Thin sections of foot tissue or plaques on Superfrost Plus slides were viewed an Olympus BX60 light microscope. Images were taken using differential interference contrast (DIC) lenses for the plaque section, and normal transmission for the foot section.

#### Secondary Ion Mass Spectroscopy (SIMS) Measurements

Tissue sections were dried on gallium arsenic wafers. Tissue was then measured with a Physical Electronics 6650. Mass gate was set at 11 Da and a charge neutralizer was used. Collection time for boron was 120 seconds.

## Results

### Mfp-5 adhesion is robust at pH 3 and lost upon exposure to pH 7.5

**Figure 2:**
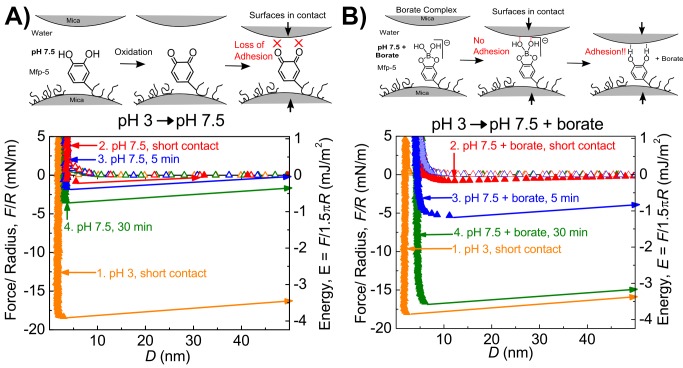
Adhesion of Mfp-5 to mica at pH 3 and pH 7.5 with and without borate. (A) Adhesion of Mfp-5 to mica at pH 3 (0.1 M HAc, 0.25 M KNO_3_) (run 1) in which buffer was increased to pH 7.5 (0.1 M Phosphate, 0.25 M KNO_3_) without borate (run 2–4). (B) Adhesion of Mfp-5 to mica at pH 3 was increased to pH 7.5 with borate (0.1 M). Boronate-complexed Mfp-5 retains nearly full adhesion. Note, overview of how to read SFA data is shown in Fig. S5. Open symbols represent the in-run.

Freshly purified Mfp-5 has nearly 30 mol% Dopa and adheres well to a variety of surface chemistries [25]. In this work, the surface forces apparatus (SFA), a device with nanoNewton force and Å distance resolution, was used to measure the adhesion forces of an Mfp-5 film compressed between two mica sheets. Cleaved mica was used because of its atomically smooth polysiloxane surface and relevance to intertidal marine geology [33]. Mfp-5 was deposited on mica to a thickness of 2 nm thick (hard wall), which is approximately equivalent to the hydrodynamic diameter of the protein (Fig. 2) [4].

After bringing the surfaces into contact, an adhesion energy (E_ad_) of −3.8 mJ/m^2^ was observed between Mfp-5 and mica (Fig. 2A), and longer times in contact resulted in E_ad_ similar to values reported elsewhere (Fig. S2) [4], [25]. Exposure of Mfp-5 to pH 7.5 reduced adhesion by 95% due to the rapid oxidation of Dopa to Dopaquinone [18]. Even extended contact times at pH 7.5 could only muster a weak E_ad_ of −0.7 mJ/m^2^. The adhesion loss was not recovered by restoring the initial pH 3.

### Retention of adhesion at neutral pH with borate

#### The Mfp-5 boronate complex maintains full adhesion at pH 7.5

**Figure 3:**
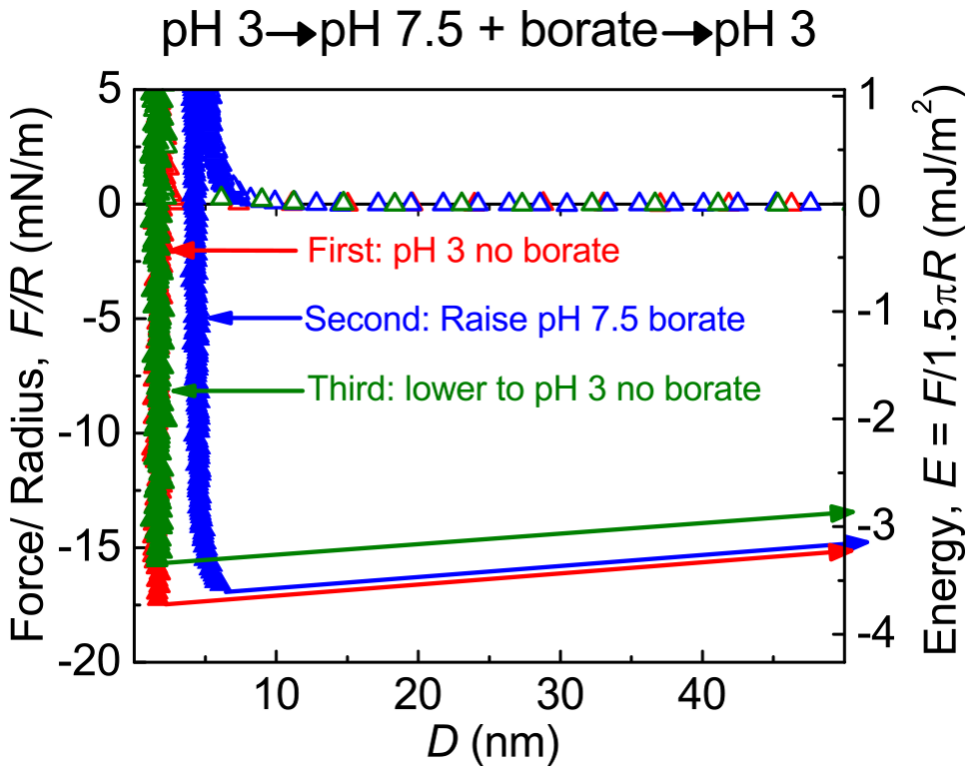
Adhesion at pH 3 after exposure to pH 7.5/borate. First, Mfp-5 adhesion at pH 3 was measured. Second, the buffer was exchanged for pH to 7.5 with borate. Third the buffer was exchanged again and returned to pH 3 with no borate.

The bidentate complexation of borate by Dopa is well known to retard oxidation. We investigated this complex to determine how Dopa-boronate complexes affect Dopa-mediated adhesion. Compared with Mfp-5′s adhesion energy at pH 3, adhesion at pH 7.5 with 100 mM borate was initially diminished by ∼95% (Fig. 2) and resembled the adhesion of Mfp-5 at pH 7.5 without borate. However, leaving surfaces in contact for a few min led to a sharp nonlinear increase in adhesion energy over time. After 30 min the E_ad_ was comparable with that observed at pH 3 after a short contact time. *The Mfp-5 boronate complex is disrupted at acidic pH*. Reversibility of Mfp-5 boronate complex formation was explored by rinsing excess borate with acetate at pH 3 (Fig. 3). The protocol of SFA treatments was as follows: adhesion energy of mfp-5 on mica was first measured at pH 3 (acetate), then measured after buffer exchange to borate at pH 7.5, and once again after a return to acetate at pH 3. During this process, Mfp-5 was exposed to pH 7.5/borate for >4 hours before returning to pH 3. The adhesion energy, hard wall, and force-distance curve measured after returning to pH 3 all matched the initial measurements at pH 3. Interestingly, adhesion properties were restored only when the SFA medium was returned to a borate-free, pH 3 buffer. Inclusion of borate anions in the rinse at pH 3 resulted in less than half the initial E_ad_ to mica (Fig. S2). Borate alone did not affect the interaction of two bare mica sheets, and XPS analysis of mica exposed to borate did not detect surface boron (Fig. S4).

#### Time-dependent adhesion of Mfp-5 boronate complexes

**Figure 4:**
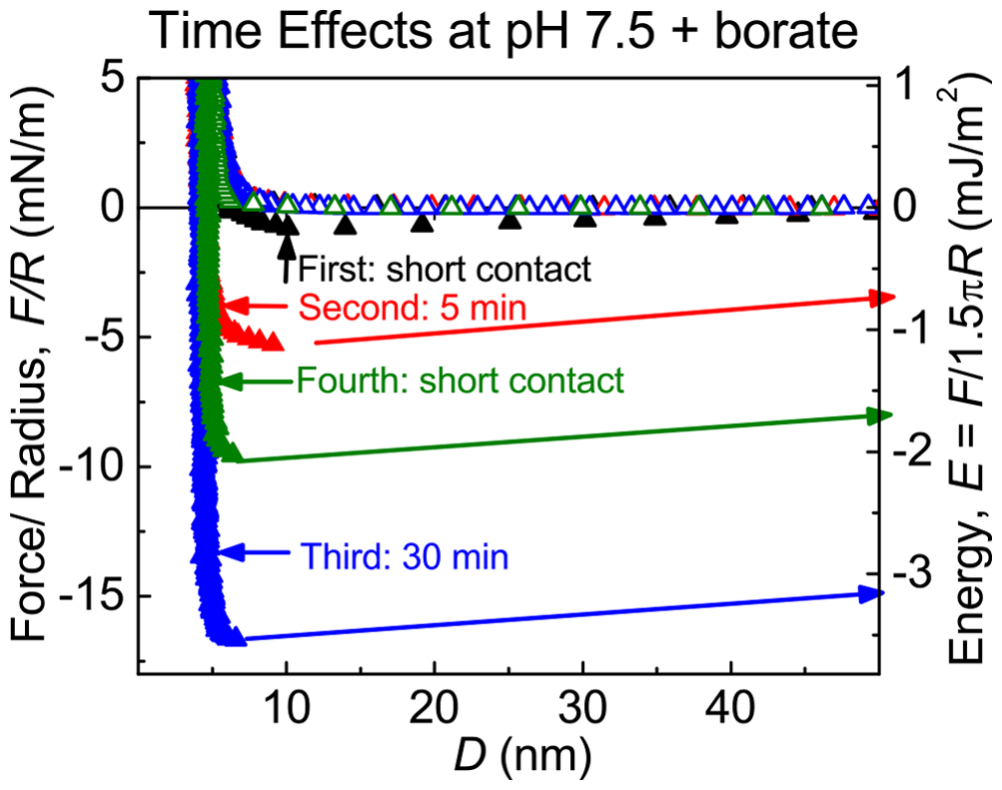
Contact-time dependence and adhesion memory of Dopa mediated Mfp-5 adhesion in borate. Surfaces were brought together briefly (<1 min) at pH 7.5 (Run 1). Contact time was then increased to 5 min (run 2) and then to 30 min contact (run 3). After the separation of run 3, the surfaces are brought back together for a shot contact (run 4). Note the difference in short contact initially vs. after 30 min contact (run 1 vs. run 4).

Mfp adhesion has been shown to be time-dependent and nonlinear before reaching an asymptotic equilibrium [4]. The adhesion of a Dopa-boronate complex is an intriguing special case in which the boronate group must dissociate before Dopa mediated adhesion is possible. Longer contact times at pH 7.5/borate were required to obtain the E_ad_ reached rapidly at pH 3 (Fig. 4). A pH 7.5/borate, the initial brief contact between Mfp-5 boronate complexes and mica resulted in negligible adhesion. Increased time in contact, however, resulted in a steep increase in E_ad_. After a 30 min contact, E_ad_ was comparable with the greatest adhesion measured at pH 3 (Figure 1B). Curiously, after a 30-min contact and separation, only a short contact time was needed for an E_ad_ of −2 mJ/m^2^. This is >15× stronger than the initial short adhesion observed at pH 7.5/borate.

#### Boron in native holdfast system

Given that borate occurs at 0.45 mM in seawater [34], it occurred to us that mussels might sequester borate for use in dispensing adhesive proteins onto surfaces. Investigation of boron distribution in the foot where Mfps are stockpiled and in adhesive plaques was done by secondary ion mass spectrometry (Fig. S1). The long collection times required to measure boron indicated little boron present. The distribution appeared uniform in both the gland and plaque, and therefore does not support the use of boron by the native mussel adhesive system.

### Boronate-Dopa dissociation enables Dopa-mica binding

#### Phenylboronic acid allows adhesion at neutral pH

**Figure 5:**
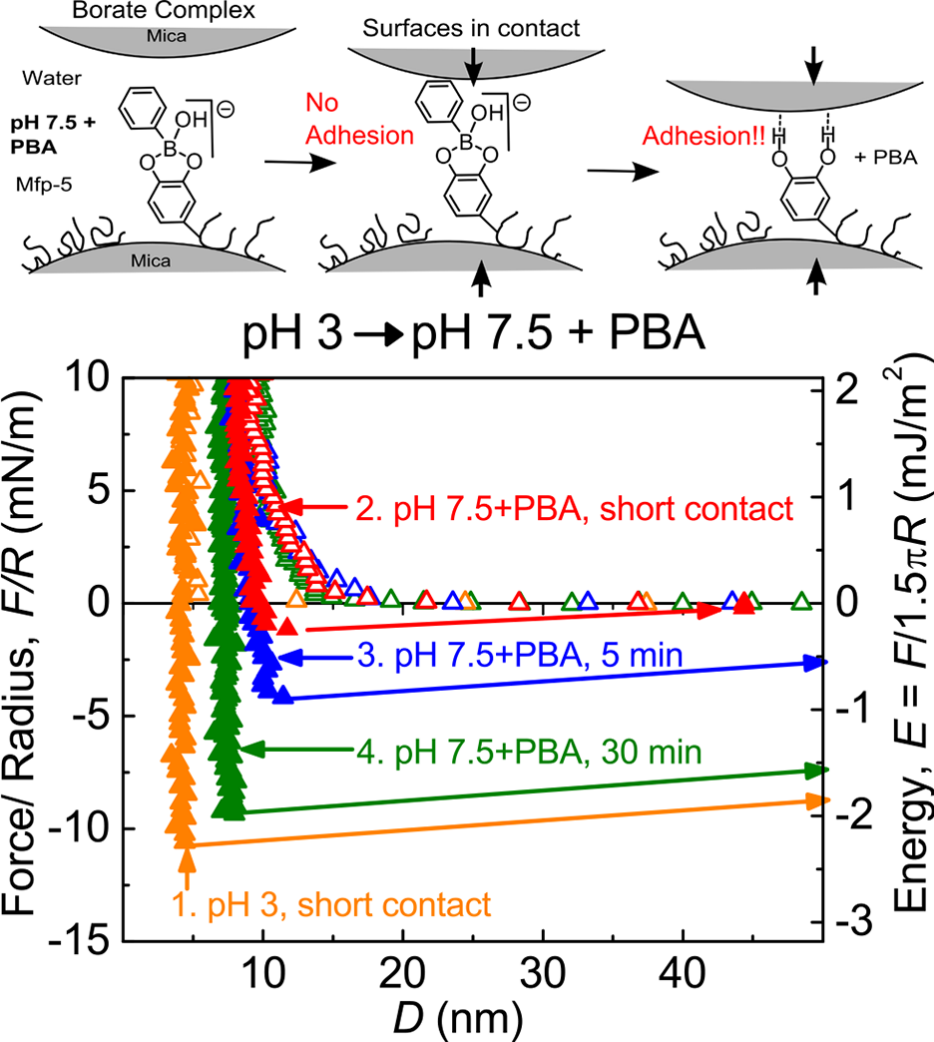
Effect of phenylboronic acid (PBA) on Mfp-5 adhesion at pH 7.5. Mfp-5 adhesion to mica was measured at pH 3 with borate free buffer. Then the buffer was exchanged to pH 7.5 with 0.1 M PBA. Surfaces are left in contact for varying times before separation.

Dopa is known to be essential for mussel foot protein adhesion to mica [3], [4], [19]. To rule out the possibility that the adhesion measured at pH 7.5/borate was due to interactions between mica and the boronate group, borate was replaced by phenylboronic acid (PBA) as the phenyl group interacts repulsively with mica [35]. In this medium, each Dopa residue in Mfp-5 forms a bidentate complex with the boronyl moiety of PBA (Fig. 5). Adhesion of PBA-Mfp-5 showed a similar time-dependence as the Mfp-5-boronate complex, with adhesion at 30 min reaching ∼90% of the level achieved at pH 3. As the phenyl group would disrupt a boronate-mediated interaction with mica and as Dopa is necessary for Mfp-5 wet adhesion, the PBA measurements support the model of borate dissociation and subsequent adhesion upon mica contact as discussed below.

#### Periodate trumps boronate protection

Previous studies have shown that the Dopa residues in Mfp-5 are effectively oxidized to Dopaquinone by periodate, a stoichiometric 2-electron oxidant. Boronate complexation shields Dopa from auto-oxidation at pH 7.5, but does it protect against directed oxidation? 20 nmol of periodate was injected into the borate solution buffer of the SFA and completely abolished adhesion (Fig. S3). Oxidation was permanent and adhesion did not recover upon returning to pH 3.

#### Adhesion of Mfp-5 in boronate complexes requires compression

**Figure 6:**
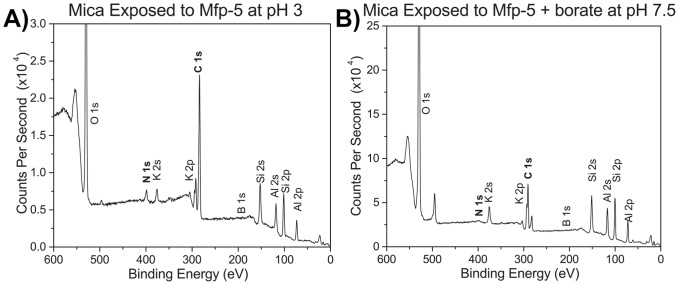
XPS spectra of mica after exposure to Mfp-5 solutions. A) Mfp-5 in pH 3 (no borate), showing an **N1s** and increased **C1s** peak due to protein adsorption from solution. Protein adsorption from solution at pH 3 is how Mfp-5 is deposited for SFA experiments. B) Mfp-5 in pH 7.5/borate solutions does not show adsorption on mica (no **N1s** peak).

Mussel adhesive plaques and purified Mfps have been shown to bind many surface chemistries [25], [36], [37]. In these systems, a compressive force is applied to enhance intimate protein-surface contact. In the SFA, compression is actuated by a motor, whereas the mussel foot relies on suction-mediated compression. To determine if Mfp-5 adsorbs to mica under force-free conditions, a droplet of Mfp-5 at pH 7.5 in borate was deposited on mica and left for 30 minutes. The surface was gently rinsed and examined by XPS for adsorbed protein (Fig. 6). Whereas pH 3 showed protein adsorption indicated by the N1s and increased C1s peaks, boronate-complexed Mfp-5 at pH 7.5 was not able to adsorb from solution to mica.

## Discussion

Oxidation control is key for effective adhesion in Dopa-containing proteins and polymers. Dopa auto-oxidation requires the deprotonation of a hydroxyl (pK_a_  = 9.3) and significant oxidation occurs even at pH 5.5 for thin films of Dopa-containing proteins [4]. The susceptibility of Dopa in Mfp-5 thin films on mica to oxidation is probably due to confinement, which alters local pK_a_, pH or both [38]. To keep Dopa-containing Mfps transiently reduced during plaque formation, the mussel is known to cosecrete an acid and antioxidants [3]. Similarly, low pH buffers and antioxidants are utilized for handling extracted mussel proteins or Dopa-containing polymers [4], [16], [19]. Dopa is stable until antioxidants, hydronium ions, or both become depleted. Redox stable synthetic analogs such as nitro-Dopa are limited in their ability to undergo curing by directed covalent cross-linking and are light-sensitive [39]. These limitations necessitate new strategies to modulate Dopa redox, both for investigating Mfps and for effective implementation of Dopa in engineered systems.

Borate complexation to Dopa, and catechols generally, has been used to avoid oxidation and form pH-responsive cross-links at pH ≥7 for drug delivery [26] and in hydrogels [26]–[28], but based on these, the effect of borate on Dopa-containing adhesives was an open-question. This work shows that the boronate complex retards oxidation without blocking the strong, reversible bonding between Mfp-5 and a mica surface (Figure 2). When the Mfp-5 boronate complex was pressed into contact with mica, a time-dependent adhesion was observed. This is in stark contrast to Mfp-5 without borate at pH 7.5, which resulted in Dopa oxidation and significant or complete adhesion loss. Multiple cycles of adhesion and separation of Mfp-5 boronate complexes with mica showed facile and reversible bonding as well as oxidation protection (Fig. 4). The rate of Dopa boronate complex formation is very fast and results in rapid protection of Dopa against oxidation, allowing reversibility [22]. Subsequent re-adhesion of Mfp-5 boronate complexes to mica was more rapid than the initial interaction with the surface (Fig. 4). Perhaps the initial interaction needs extra time to overcome the coulombic repulsion with the negatively charged mica surface which, once achieved, becomes easier to replicate despite the adhesive failure. The results suggest that Dopa-boronate functionalities could rapidly bond surfaces if engineered to have a favorable conformation.

Adhesion of Mfp-5 to mica was previously proposed to be Dopa-mediated [4], [19]. Dopa mediation is also confirmed in this work by the reduced E_ad_ observed after exposure to neutral pH (Fig. 2) and by periodate-induced oxidation (Fig. S3). Given these, the Mfp-5 boronate complex poses an obstacle to Dopa-mediated adhesion in that a prior dissociation to Dopa and borate is required. As previously noted, borate and Dopa form a boronate anion having a weak stability constant. Electron withdrawing groups on boron are reported to stabilize the complex, whereas electron donating groups destabilize it [22], [23]. We propose that the negative surface charge of mica at pH 7.5 destabilizes the boronate complex. The interaction of Dopa-boronate anions with the negatively charged surface induces dissociation, freeing the Dopa for surface binding. Borate, alone, was shown by XPS and SFA not to bind mica surfaces (Fig. S4). The unlikely scenario of mica surface bonding mediated by the boronate group was ruled out by use of PBA, that is, a phenyl group would disrupt any such interaction (Fig. 5) [35]. Dissociation upon contact with a negatively charged surface implies the borate protection strategy should be widely applicable, as most surfaces are negatively charged at pH 7.5 (e.g. titania, silica, and various iron oxides [40]–[42]).

Mfp-5 is known to adhere well to many surfaces [25]. A high E_ad_ can help overcome initial repulsive forces such as the electrostatic double layer force near a material's surface. SFA studies of Mfp-5 forced into contact with a polystyrene (PS) surface showed adhesion [25], whereas Overhauser Dynamic Nuclear Polarization (ODNP) measurements did not detect adsorption of Mfp-5 on spin-labeled PS beads [43]. At pH 7.5, Mfp-5 binds to mica when under compression in the SFA (Fig. 2), but does not adsorb under force-free conditions (Fig. 6). In summary, for effective use of borate-buffered Dopa in adhesion, load may be required just as a glued joint requires clamping.

Catechol boronate complexes are widely used to protect catechols from oxidation at neutral pH or to form a pH-responsive bond. A complete switch in buffering solution from pH 7.5/borate to pH 3 resulted in an adhesion energy that matched the initial adhesion of Mfp-5 at pH3 and indicated complete adhesion preservation by borate (Fig. 3). However, when borate was added to an acidic solution of Mfp-5 or when pH 7.5/borate was exchanged with pH 3 without a complete rinse, residual borate reduced adhesion by ∼60% (Fig. S2). This is an important caveat for the effective use of borate protection in Dopa-containing adhesives. Understanding the unexpected interference of borate on adhesion at acidic pH requires further work.

pH responsive self-healing hydrogels based on catechol boronate complexation have been prepared [27], [28], [44], [45]. The complexation between borate and catechols is promising as the B-O- covalent bond is pH responsive. Borate itself is a micronutrient [34] with a very low toxicity (LD50 = 1.3 g/kg [46]). Our measurements have found effective Dopa protection and adhesion at 1mM borate (12 mg/kg). As effective implementation of hydrogels requires adhesion to a substrate, catechol-boronate stimuli-responsive hydrogels may prove to be well suited for adhesion as well as stimuli-responsiveness.

## Conclusion

As applications exploiting the versatile reactivity of Dopa in proteins and polymers continue to be expanded, it is increasingly important to find ways to control Dopa's redox activity. This study shows the effectiveness of borate in controlling Dopa oxidation at neutral pH, while still allowing for adhesion. Borate combines with Dopa to produce a pH-dependent, reversible, and bidentate Dopa-boronate bond that resists oxidation. Mfp-5 boronate complexes stabilize redox as well as enabling strong, reversible, Dopa-mediated adhesion to mica at neutral pH. This discovery will enable Dopa functionalized adhesives to be used under conditions that would otherwise void Dopa's utility.

## Supporting Information

Figure S1 **Boron distribution in the mussel phenol gland and adhesive plaque. (**A) An overview of mussel anatomy showing locations of the foot (B) and plaque (C). (B) A mussel foot section containing the horseshoe shaped phenol gland where Mfp-5 is stockpiled. Box indicates location of boron measurements by SIMS. Scale bar: 500 µm for LM and 60 µm for SIMS. (C) A section of an adhesive plaque viewed with DIC and measured for boron by SIMS. Scale bar: 500 µm for LM and 60 µm for SIMS.
(TIF)

Figure S2 **Mfp-5 adhesion at pH 3 with and without borate.** (A) Adhesion of Mfp-5 on mica at pH 3, 0.1 M acetic acid buffer, no borate. (B) The same setup was then rinsed with pH 3, 0.1 M acetic acid buffer with 0.1 M borate. Force runs are done with various contact times. Note Y-axis change. Open symbols represent the in-run.
(TIF)

Figure S3 **Measurement of Mfp-5 adhesion before and after addition of the oxidant periodate.** Mfp-5 deposited onto mica shows adhesion at pH 7.5, 0.1 M phosphate, 0.25 M KNO_3_, 0.1 M borate (First). Mfp-5 was then reacted with 20 nmol of periodate (Second). A buffer exchange to pH 3, 0.1 M acetic acid was unable to restore adhesion (Third).
(TIF)

Figure S4 **Boron interaction with mica. (**A) XPS spectra of mica surfaces exposed to a 0.1 M borate solution in both pH 7.5, 0.1 M PBS and pH 3, 0.1 M acetic acid. There is no signal of boron on the surface. (B) SFA measurement of mica-mica interactions in the various solution conditions used in this study. No mica-mica adhesion was found at pH 7.5, and pH 3 showed weak adhesion.
(TIF)

Figure S5 **Guide for SFA data interpretation. An example run of Mfp-5 in pH 7.5/borate with a 30 min contact.** (1) The surfaces approach. (2) The surfaces meet and cannot come closer. This is the “hard-wall” as increased force does not compress the layer further. The motor is then halted and the surfaces are left in contact. (3) After 30 minutes, a separation force is applied and the surfaces are initially held together by Mfp-5. (4) The E_ad_ is overcome and a rapid separation between the surfaces is experienced, a “jump-out”.
(TIF)
