# Supplementary figures and images for: Boronate Complex Formation with Dopa Containing Mussel Adhesive Protein Retards pH-Induced Oxidation and Enables Adhesion to Mica
Source: PLoS One. 2014 Oct 10;9(10):e108869. doi: 10.1371/journal.pone.0108869 (PMC4193769; doi:10.1371/journal.pone.0108869)

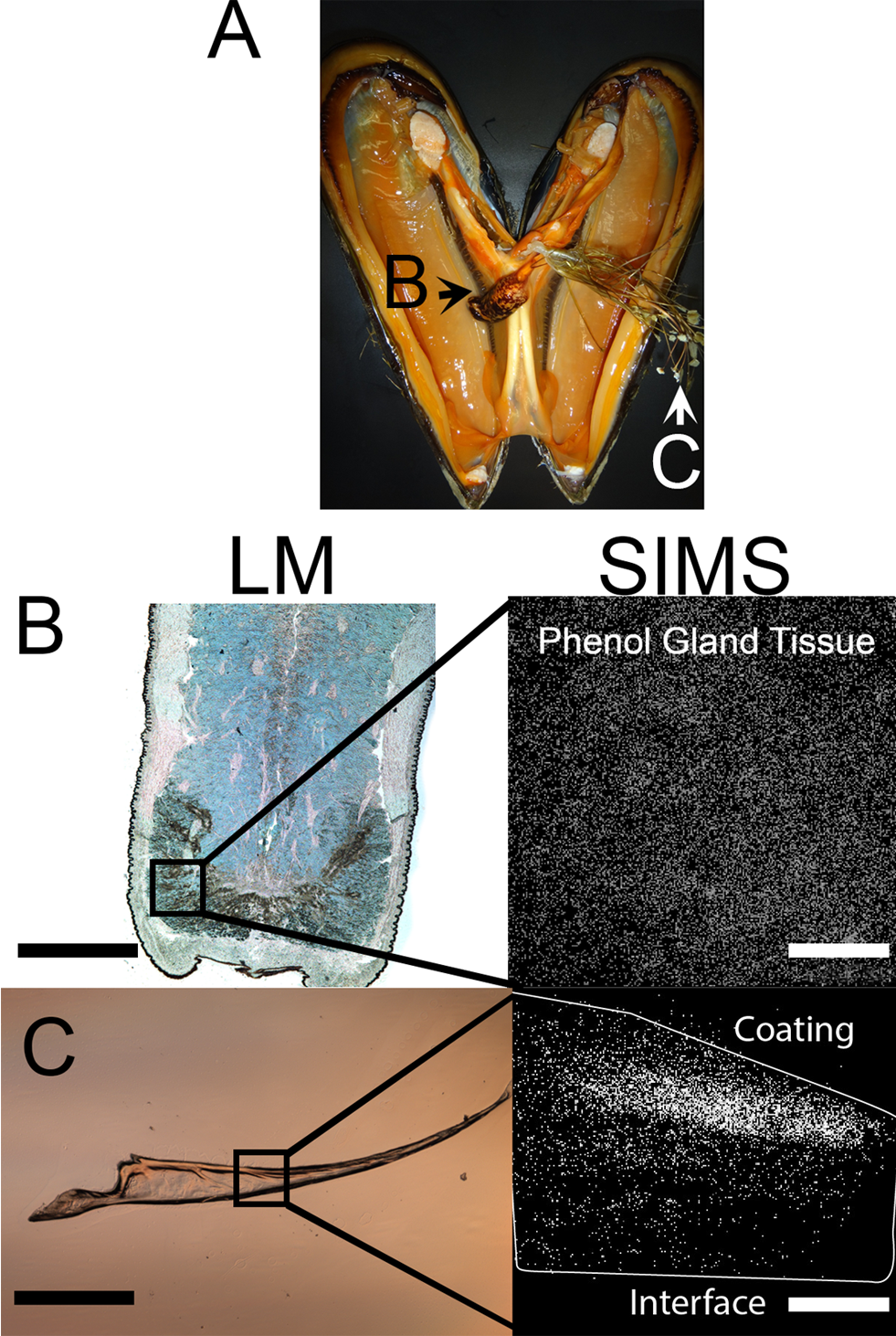

Supplement: Figure S1 — Boron distribution in the mussel phenol gland and adhesive plaque. (A) An overview of mussel anatomy showing locations of the foot (B) and plaque (C). (B) A mussel foot section containing the horseshoe shaped phenol gland where Mfp-5 is stockpiled. Box indicates location of boron measurements by SIMS. Scale bar: 500 µm for LM and 60 µm for SIMS. (C) A section of an adhesive plaque viewed with DIC and measured for boron by SIMS. Scale bar: 500 µm for LM and 60 µm for SIMS. (TIF) [file pone.0108869.s001.tif]

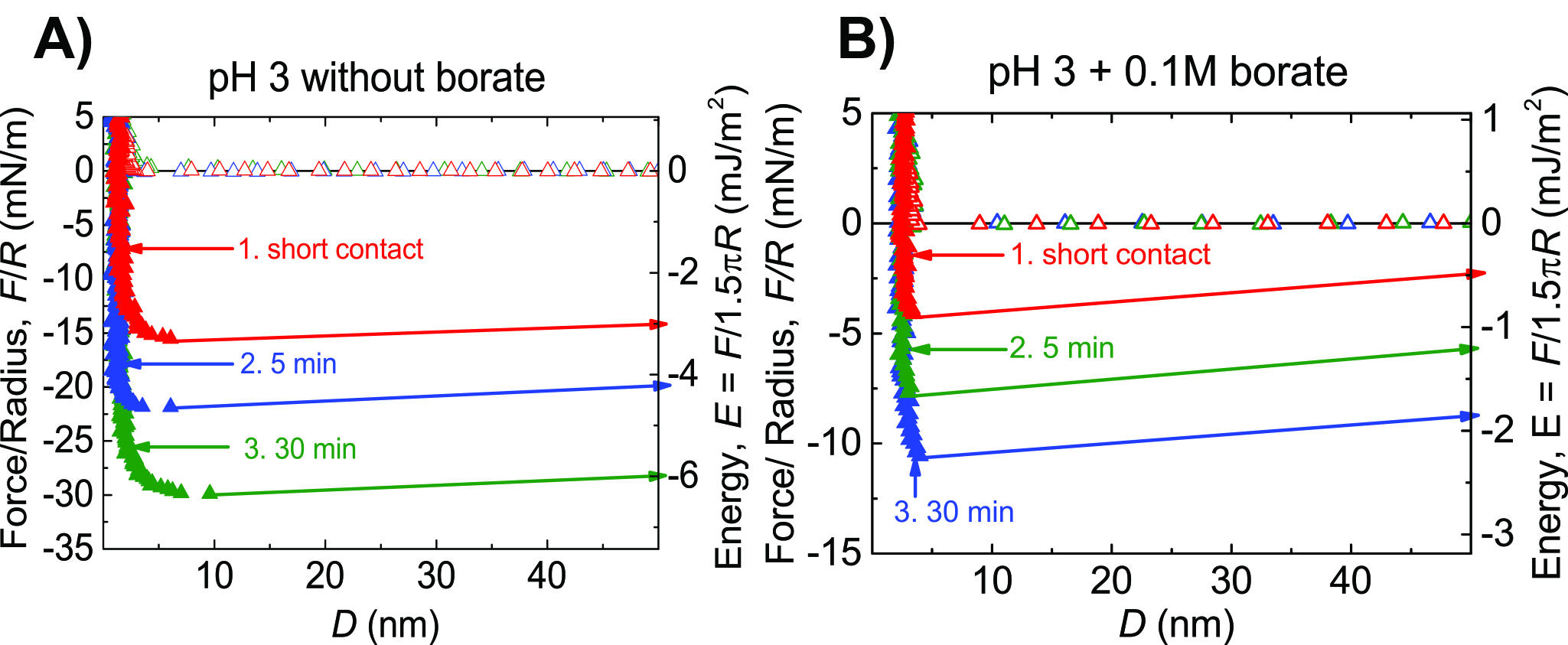

Supplement: Figure S2 — Mfp-5 adhesion at pH 3 with and without borate. (A) Adhesion of Mfp-5 on mica at pH 3, 0.1 M acetic acid buffer, no borate. (B) The same setup was then rinsed with pH 3, 0.1 M acetic acid buffer with 0.1 M borate. Force runs are done with various contact times. Note Y-axis change. Open symbols represent the in-run. (TIF) [file pone.0108869.s002.tif]

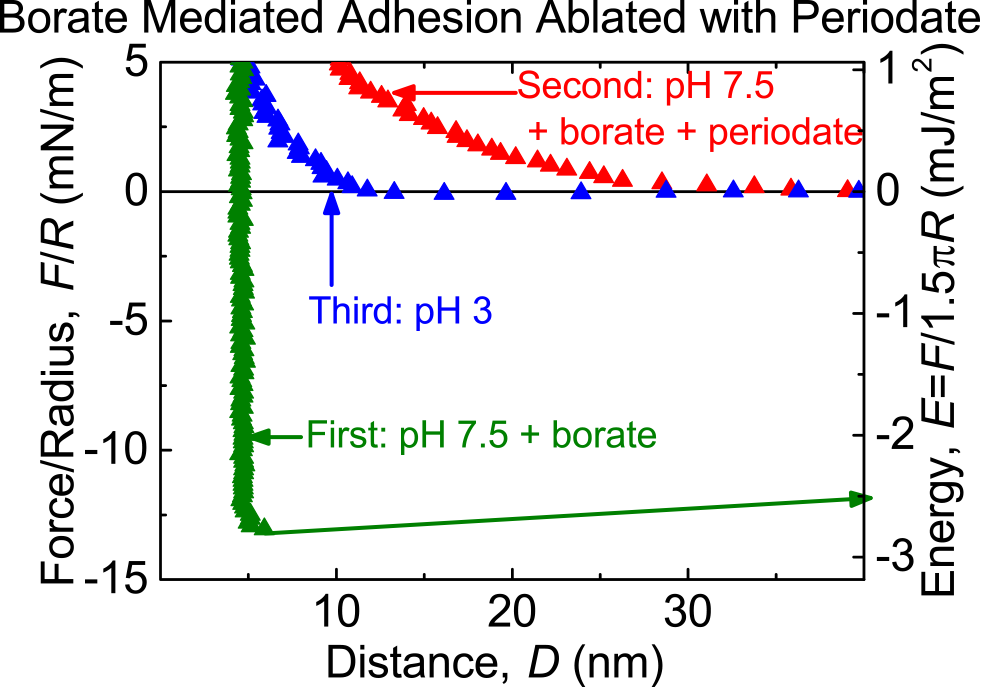

Supplement: Figure S3 — Measurement of Mfp-5 adhesion before and after addition of the oxidant periodate. Mfp-5 deposited onto mica shows adhesion at pH 7.5, 0.1 M phosphate, 0.25 M KNO3, 0.1 M borate (First). Mfp-5 was then reacted with 20 nmol of periodate (Second). A buffer exchange to pH 3, 0.1 M acetic acid was unable to restore adhesion (Third). (TIF) [file pone.0108869.s003.tif]

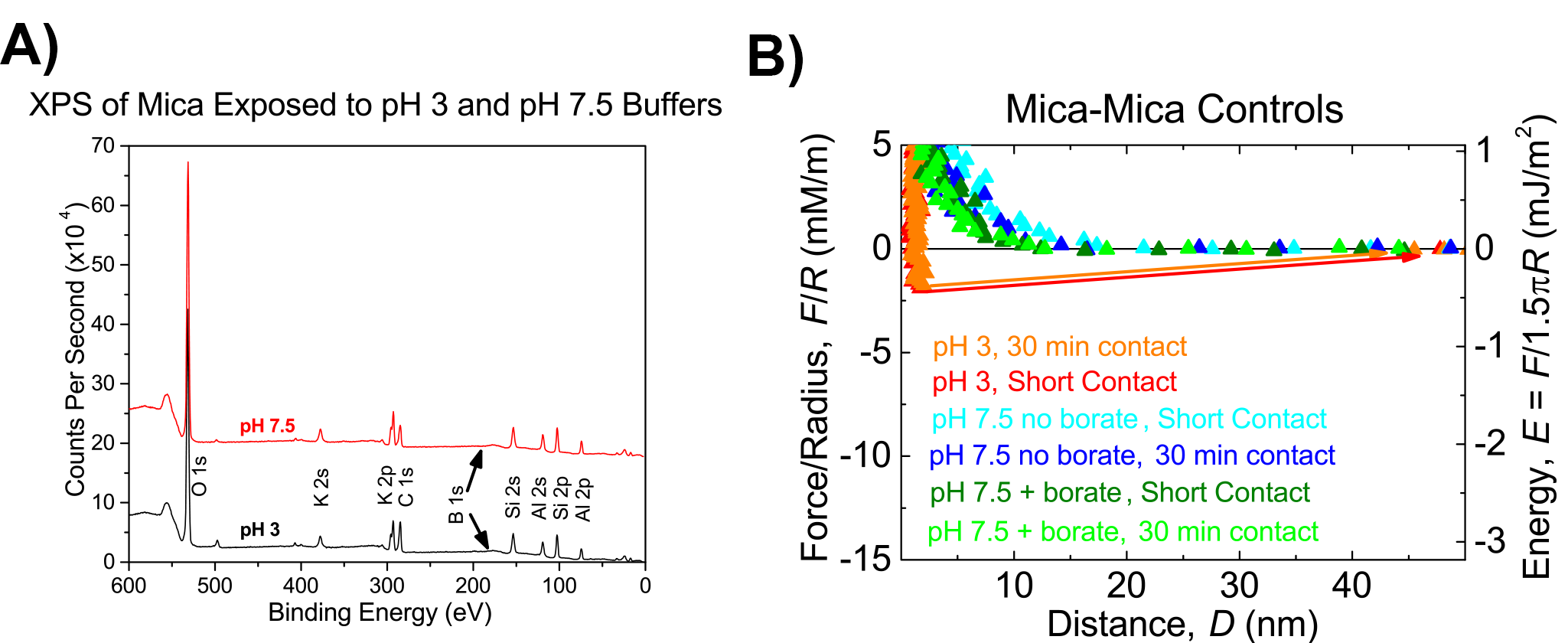

Supplement: Figure S4 — Boron interaction with mica. (A) XPS spectra of mica surfaces exposed to a 0.1 M borate solution in both pH 7.5, 0.1 M PBS and pH 3, 0.1 M acetic acid. There is no signal of boron on the surface. (B) SFA measurement of mica-mica interactions in the various solution conditions used in this study. No mica-mica adhesion was found at pH 7.5, and pH 3 showed weak adhesion. (TIF) [file pone.0108869.s004.tif]

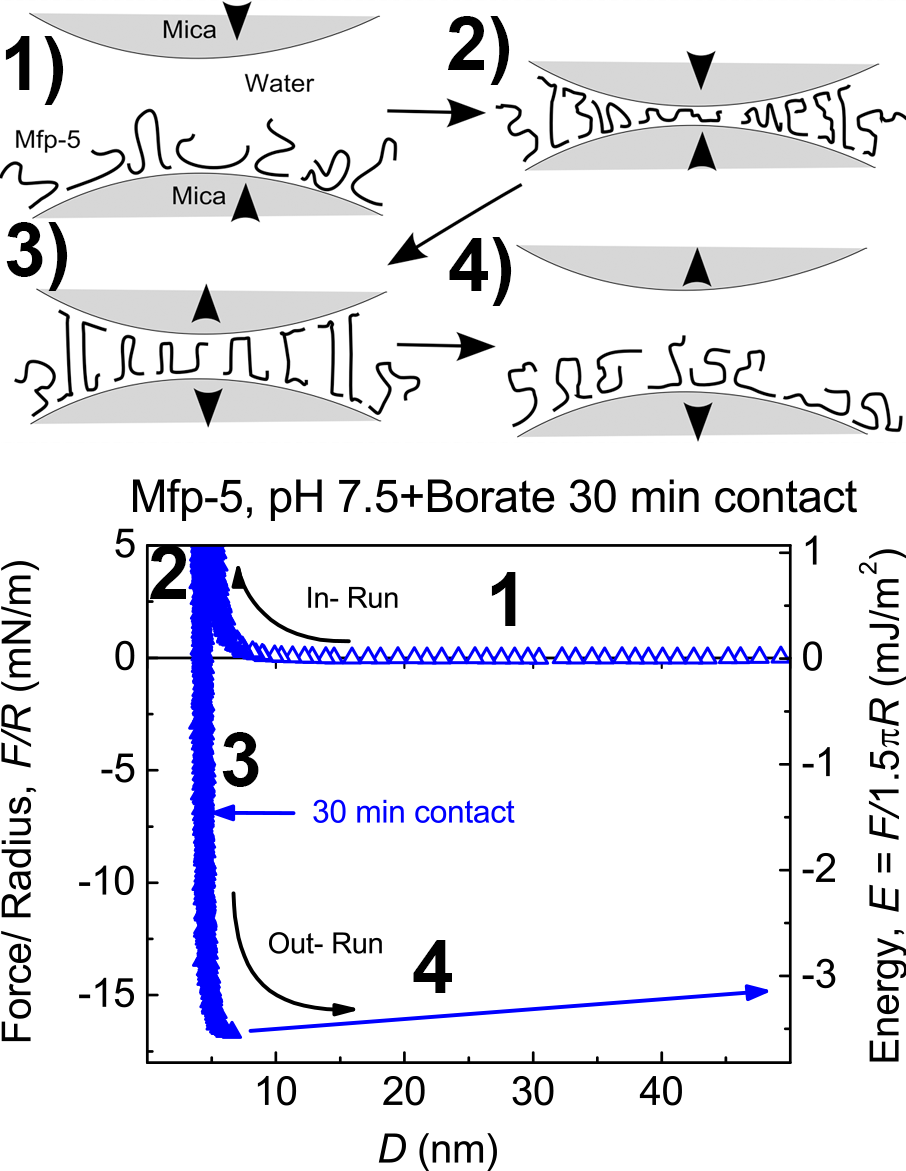

Supplement: Figure S5 — Guide for SFA data interpretation. An example run of Mfp-5 in pH 7.5/borate with a 30 min contact. (1) The surfaces approach. (2) The surfaces meet and cannot come closer. This is the “hard-wall” as increased force does not compress the layer further. The motor is then halted and the surfaces are left in contact. (3) After 30 minutes, a separation force is applied and the surfaces are initially held together by Mfp-5. (4) The Ead is overcome and a rapid separation between the surfaces is experienced, a “jump-out”. (TIF) [file pone.0108869.s005.tif]
